# Cradle of rebellion: the relationship between bone marrow microenvironment and autoimmune diseases

**DOI:** 10.3389/fimmu.2026.1760577

**Published:** 2026-10-22

**Authors:** Qian Wang, Jiashuo Cheng, Zhifeng Gu, Chen Dong

**Affiliations:** Department of Rheumatology, Research Center of Clinical Medicine, Research Center of Clinical Immunology, Affiliated Hospital of Nantong University, Medical School of Nantong University, Nantong University, Nantong, China

**Keywords:** autoimmune diseases, bone marrow microenvironment, hematopoietic stem cells, immunomodulation, mesenchymal stromal cells

## Abstract

Abnormalities in bone marrow microenvironment (BMM) represent a key mechanism closely related to the occurrence and progression of autoimmune diseases (AIDs). They involve dysfunction of critical microenvironment components: reduced supportive function of mesenchymal stromal cells (MSCs) leads to decreased secretion of the anchoring factor CXCL12, disrupting the quiescent state of hematopoietic stem cells (HSCs); dysregulation of sympathetic tone disturbs the circadian rhythm of hematopoietic cell arrangement; and abnormal proliferation of adipocytes fosters a pro-inflammatory environment. Collectively, these changes are related to a functional shift in the microenvironment from homeostasis maintenance to inflammation promotion, directionally biasing HSCs towards differentiation into pro-inflammatory immune lineages and facilitating massive release into the periphery. It establishes a pathogenic vicious cycle: peripheral autoimmune inflammation feeds back to the bone marrow (BM), further remodeling the microenvironment and perpetuating the production of aggressive immune cells, including autoreactive T cells, autoantibody-producing cells, and pro-inflammatory myeloid cells. As a result, patients experience a persistent inflammatory state that resists complete resolution, and disease progression is characterized by systemic involvement, chronicity, and relapsing course. This review focuses on the cellular and molecular components, functional roles, pathological alterations, and mechanisms of the BMM in AIDs. The review aims to provide an essential foundation for further investigation into the pathogenesis of autoimmune diseases.

## 1 Introduction

Autoimmune diseases (AIDs) encompass a spectrum of chronic inflammatory diseases characterized by the immune system’s loss of tolerance to self-antigens. AIDs can be broadly categorized into organ-specific diseases, such as Graves’ disease, type 1 diabetes mellitus (T1DM) and autoimmune liver disease; and systemic autoimmune diseases, such as multiple sclerosis (MS), antiphospholipid syndrome (APS), rheumatoid arthritis (RA) and systemic lupus erythematosus (SLE) (1, 2). The pathogenesis of AIDs involves the dual defects of central and peripheral tolerance. As a key immune organ, the bone marrow (BM) plays an important role: it serves as the site for B lymphocyte development and initial lymphocyte output, while also providing a specialized microenvironment for long-lived plasma cells (LLPCs) and memory T cells. The bone marrow microenvironment (BMM) constitutes a complex and dynamic microenvironment, composed of diverse cellular populations, cytokines, chemokines, and extracellular matrix. These elements function in concert to orchestrate the development and differentiation of HSCs and immune cells, thereby playing a critical role in maintaining overall health (3). As both the birthplace of immune cells and a key site for regulating immune response and tolerance (4), the BMM is fundamental to immune homeostasis. Under physiological homeostasis, the BMM maintains quiescence and self-renewal ability through tightly regulated molecular signaling pathways. However, upon exposure to acute and chronic stress, the microenvironment undergoes compositional and functional alterations across its cellular compartments, structural remodeling and signal dysregulation (5–7). These pathological changes directly impair its ability to maintain immune tolerance, leading to aberrant activation or escape of autoreactive immune cells that ultimately attack the host tissues (**Figure 1**). This microenvironmental perspective provides a mechanistic explanation for the breakdown of autoimmune tolerance at the microenvironment level.

**Figure 1:**
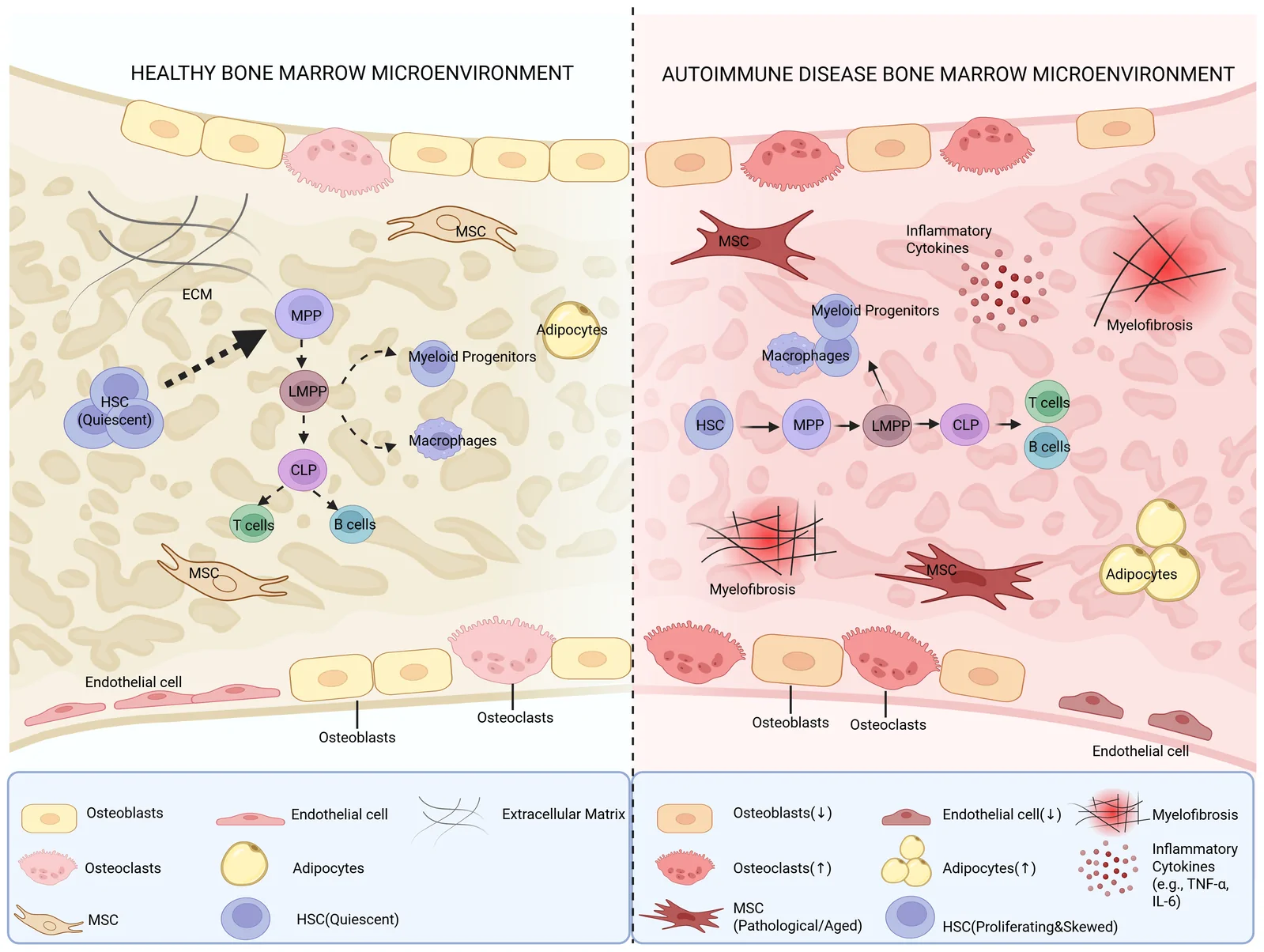
Comparison of BMM between homeostatic and autoimmune conditions.

Given the BMM’s foundational role in immune homeostasis, dissecting how it transitions from the cradle of immune homeostasis to the focus of autoimmunity is crucial for understanding disease pathogenesis. This review systematically characterizes the abnormal alterations of the BMM in autoimmune diseases, and explores the potential links between microenvironment dysregulation and disease onset/progression from the perspectives of cellular components, molecular mechanisms, and clinical translational significance, so as to provide a theoretical foundation for the development of treatment strategies centered on targeting BMM and reconstructing immune tolerance.

## 2 Structural composition and homeostatic regulatory function of bone marrow microenvironment

In terms of cellular composition and architecture, the BMM comprises both hematopoietic and non-hematopoietic compartments. The hematopoietic compartment includes HSCs, multipotent progenitor cells, precursor cells, and mature immune cells. The non-hematopoietic stromal compartment consists of mesenchymal stromal cells (MSCs) with multilineage differentiation potential, osteolineage cells (such as osteoblasts) that constitute the endosteal niche, and vascular niche cells (including vascular endothelial cells and pericytes) (8–10). Adipocytes are also present, contributing to energy metabolism and immune regulation (11). The extracellular matrix, which is composed of collagen, fibronectin, laminin, and other components, provides structural support and mediates signal transduction, alongside a complex array of cytokines and neuroendocrine signals (12). BMM mainly consists of arterial-endosteal niches, sinusoidal niches, adipocyte-associated niches and non-vascular mixed-lineage niches. Among them, the sinusoids, as the most widely distributed vascular structure, form a reticular network throughout the BM, mainly located in the central medullary cavity and adjacent to mature myeloid cells, suggesting that they serve as a channel for mature blood cells to be released into the bloodstream. Surrounding the sinusoids are Lepr^+^ Adipo-MSCs (adipocyte-related MSCs with high expression of leptin receptor), which also highly express hematopoietic support factors such as CXCL12 and KITLG, and are the core functional cells of the sinusoidal niches and adipocyte-associated niches. HSCs have a close spatial association with sinusoids and Lepr^+^ Adipo-MSCs. Lepr^+^ Adipo-MSCs maintain the stemness of HSCs by secreting key factors, while sinusoids provide migration and survival support for HSCs. Early myeloid progenitors and granulocyte-monocyte progenitors are mainly distributed in arterial-endosteal niches, and mature myeloid cells exit in sinusoidal niches. Together, these structural and functional elements ensure the precise regulation of hematopoiesis and immune homeostasis (13, 14).

The BMM serves as the cradle for the orderly development of immune cells and exhibits a high degree of functional plasticity. Under steady-state conditions, its primary role is to maintain HSC quiescence and self-renewal. As the common origin of all immune cells, HSCs within the BMM first differentiate into multipotent progenitor cells (MPPs), which subsequently generate lymphoid-primed multipotent progenitors (LMPPs). These cells have lost the ability to adopt erythroid and megakaryocyte lineage fates, but sustain a high proliferative and combined lympho-myeloid differentiation potential. Common lymphoid progenitors (CLPs) are developmentally downstream of LMPPs, and their differentiation potential is mainly restricted to the lymphoid lineage (15, 16). In adult BM, HSCs remain quiescent most of the time, with their proliferation and differentiation tightly regulated by the BMM to sustain lifelong hematopoietic homeostasis. However, under stress conditions such as infection or inflammation, the microenvironment drives HSCs to exit quiescence and enhances proliferation, particularly skewing differentiation toward the myeloid lineage, to meet physiological demands. Nevertheless, prolonged or persistent inflammatory stimulation can impair hematopoietic stem and progenitor cell (HSPC) function (8, 17, 18).

The BMM plays a critical role in maintaining both central and peripheral immune homeostasis. Within the central tolerance mechanism, for instance, immature B cells that encounter self-antigens undergo clonal deletion, receptor editing, or functional inactivation if their B cell receptors (BCRs) bind with high affinity to self-antigens. This process is completed in the BMM (19). Despite these precise mechanisms, a small number of autoreactive lymphocytes may still escape into the periphery. Under normal physiological conditions, it does not lead to autoimmune pathology, which can be called physiological autoimmunity (2). However, once the BMM homeostasis itself is disrupted, its ability to induce central tolerance becomes impaired, resulting in increased escape of autoreactive cells into the periphery, which in turn contributes significantly to the initiation and progression of autoimmune diseases (20). In summary, the intricate interactions among the cellular and molecular components of the BMM collectively uphold its homeostatic balance, ensuring the proper execution of hematopoietic and immune processes across the lifespan.

## 3 Pathological manifestations of BMM in AIDs

Traditionally, studies on AIDs have focused predominantly on peripheral lymphoid organs and target tissues (such as the synovium in RA), while the role of the BM has long received insufficient attention. However, recent studies have shown that a variety of AIDs exhibit significant abnormalities in the BMM. These pathological alterations encompass functional decline of MSCs, impaired hematopoietic support capacity, biased immune cell development, bone remodeling imbalance, establishment of a pro-inflammatory microenvironment, and metabolic disorders (**Table 1**). These findings highlight the BM as an active agent of autoimmune pathology. This chapter systematically summarizes current research on the abnormal changes and characteristics of the BMM in AIDs, highlighting its emerging role as a critical player in disease pathogenesis.

**Table 1:** Changes in BMM components in AIDs.

Component	Normal function	Pathological alteration in AIDs	Consequence	Reference
Mesenchymal stromal cells (MSCs)	Maintain HSC quiescence via CXCL12, immunomodulation, differentiate into osteoblasts/adipocytes	Senescence (SLE), reduced CXCL12, reduced osteogenic differentiation (SLE, IBD), increased adipogenic differentiation (IBD), impaired immunosuppression	Disrupted HSC anchoring, enhanced inflammatory responses, biased differentiation	(37, 43, 52, 55–57)
Hematopoietic stem cells (HSCs)	Self-renewal, multilineage differentiation, quiescence (mainly anaerobic glycolysis)	Metabolic reprogramming (oxidative phosphorylation ↑), ROS accumulation, DNA damage, myeloid bias, exhaustion	Impaired hematopoiesis, pro-inflammatory immune output	(62–64)
Vascular endothelial cells	Form vascular niche, regulate HSC trafficking via E-selectin, secrete SCF/CXCL12	Dysfunction, impaired regeneration (T1DM)	Disrupted HSC homing and mobilization	(76–79, 117)
Adipocytes	Energy metabolism, under homeostasis, low abundance	Excessive adipogenesis (IBD), increased BM fat infiltration (IBD)	Pro-inflammatory environment, impaired hematopoiesis	(52, 82, 84)
Cytokine network	Maintain immune balance	Elevated pro-inflammatory cytokines (TNF-α, IFN-γ), chronic immune activation, feedback amplification	Sustained HSC activation, immune dysregulation, establishment of inflammatory microenvironment	(72, 113)
Neural regulation	Control circadian HSC mobilization and niche signaling via sympathetic nerves	Disrupted sympathetic signaling and circadian rhythm	Altered HSC trafficking, impaired niche coordination, exacerbation of microenvironmental instability	(85, 86, 89)

### 3.1 Structural abnormalities

AIDs are fundamentally characterized by the disruption of immune homeostasis. In recent years, many studies have demonstrated that the BMM of these conditions exhibits multiple structural alterations, including vascular dysfunction, fibrous replacement of normal hematopoietic tissue, and changes in composition, abundance, and function of adipocytes (21). For example, in autoimmune hemolytic anemia (AIHA), the BMM displays reticulin fibrosis, increased pathological cell infiltration, disordered erythropoiesis, an imbalanced cytokine network and abnormal T lymphocyte infiltration (22, 23). Bone marrow fibrosis has also been clearly documented in the case reports of SLE (24–27). SLE patients may present with varying degrees of marrow fibrosis, and clinical observations indicate that improvement in SLE disease activity correlates with amelioration of pancytopenia, suggesting that effective immunosuppressive therapy may reduce fibrosis and restore BM function (28–30). In IgG4-related disease (IgG4-RD), as a rare immune-mediated condition distinct from malignancy and infection, the BM has been identified as an important affected organ. It has been proven that there is extensive infiltration of IgG4-positive plasma cells in the BM of the patients, and PET-CT shows numerous hypermetabolic lesions within the BMM (31, 32). The damage to the physical architecture and the chemical signaling environment of the BM disrupts the normal spatial interactions and functional communication between stem cells and microenvironmental cells. This ultimately hampers the proper differentiation and production of immune cells, fostering an environment that promotes dysfunctional immune cell emergence and perpetuates a self-sustaining pathological cycle.

In addition, a close and bidirectional relationship exists between BM damage and bone remodeling in RA. RA is a systemic chronic autoimmune disease primarily characterized by destruction of joint synovial tissue, frequently accompanied by extra-articular manifestations including skin involvement (rheumatoid nodules), vasculitis, and hematological abnormalities such as anemia (33). Within the BMM of RA patients, a state of hyperactivation driven by systemic inflammation is consistently observed. On the one hand, inflammatory CD4^+^ T cells, particularly Th17 cells, are significantly increased in the BM. On the other hand, long-lived autoantibody producing cells reside within the BM of RA patients, serving not only as the persistent source of serum anti-citrullinated peptide antibodies (ACPA), but also potentially contributing directly to disease pathology through mechanisms such as neutrophil targeting (34, 35). BM edema is a common feature in RA patients, reflecting intense inflammatory cell infiltration within the marrow cavity and abundant production of pro-inflammatory cytokines. This inflammatory milieu drives the expansion and recruitment of osteoclast precursors with enhanced osteoclastic potential. Under chronic inflammatory conditions, these precursors, located in both the BM and synovial microenvironment, are continuously stimulated by RANKL derived from multiple cellular sources, leading to their differentiation into mature osteoclasts and forming active bone resorption foci beneath the bone cortex, directly mediating the development of bone erosion (36). Therefore, BM edema in RA is regarded as the direct precursor of bone erosion. The persistence of the inflammatory state of the BMM is, therefore, closely related to the imbalance in bone remodeling and gradual bone loss that is typical of this disease.

### 3.2 Impaired MSCs

MSCs are essential stromal components of the BMM. As precursor cells with self-renewal capacity, they can differentiate into chondrocytes, osteoblasts, and adipocytes, positioning them as core regulators of BM homeostasis (37, 38). Under pathological conditions, MSCs are continuously exposed to inflammatory cytokines such as IFN-γ and TNF-α, which impair their normal immunosuppressive function and disrupt their immunomodulatory balance. This dysregulation promotes local immune and inflammatory responses through multiple mechanisms, exacerbating the inflammatory storm within the BMM (39–41). Moreover, abnormally activated MSCs may participate in the process of antigen presentation and immune activation, inhibiting effector T cells in an antigen-specific manner while paradoxically forming focal inflammatory microenvironments that aggregate lymphocytes and disrupt immune homeostasis (42). The CXCL12/CXCR4 axis, primarily maintained by MSCs, represents the most critical signaling pathway for anchoring HSCs within the microenvironment, directly influencing HSC mobilization and homing (43). In addition, MSCs tightly regulate other essential signaling pathways that govern HSC fate. Stem cell factor (SCF)/c-kit axis is essential for the survival and maintenance of HSCs, and MSCs serve as the main source of both membrane-bound and soluble SCF in the BMM. By expressing Jagged1 and other Notch ligands, MSCs trigger the Notch signaling cascade, thereby fostering the self-renewal of hematopoietic stem cells. Furthermore, MSCs synthesize and secrete a broad spectrum of Notch and Wnt related signaling molecules, including Notch receptors and ligands, as well as Wnt ligands. Through these signaling cascades, MSCs engage in reciprocal crosstalk with hematopoietic stem cells and leukemic cells alike, modulating core HSC functions such as self-renewal, lineage differentiation, and homing (44–48). By releasing norepinephrine, the sympathetic nervous system modulates MSC proliferation and CXCL12 secretion in a circadian rhythm-dependent manner, which underlies the diurnal fluctuations observed in HSC mobilization from BM to peripheral blood (49). As key regulatory cellular elements within the BMM, MSCs play a significant role in the progression of various AIDs.

Autoimmune liver disease (AILD) encompasses autoimmune hepatitis (AIH), primary biliary cholangitis (PBC) and primary sclerosing cholangitis (PSC), classified according to the primary target cells. The pathogenesis of AILD is primarily driven by autoreactive T cell-mediated cytotoxicity, extensive plasma cell infiltration, and production of multiple autoantibodies (50). Studies have revealed distinct BM abnormalities, which are mostly secondary changes caused by chronic inflammation and nutritional deficiency, across different AILD subtypes (51). In PBC patients, HSPCs and MSCs exhibit significant numerical and functional defects. In contrast, AIH patients demonstrate a highly activated or proliferative BM state with markedly increased HSPCs, while bone marrow stromal cells retain their capacity to support normal hematopoiesis.

Inflammatory bowel disease (IBD) similarly demonstrates that systemic inflammatory states directly impair BM stromal cell function. The inflammatory microenvironment within the BM induces downregulation of osteogenic genes and upregulation of adipogenic genes in MSCs, shifting their differentiation preference from osteogenesis toward adipogenesis. This lineage skewing ultimately results in reduced bone mass and increased fat infiltration in BM (52). Compared to AILD, IBD-related BM involvement appears more direct and central to pathogenesis. The marrow-gut axis drives homing of aberrant immune cells from intestinal inflammatory sites to the BM via the bloodstream. The upregulation of Elf-1 and key molecules such as the deubiquitinating enzyme OTUD6A disrupts immune cell differentiation and maturation within the BMM and amplifies inflammatory signals locally (53). Upon trafficking from the intestinal inflammatory environment and homing to the marrow, these aberrant immune cells establish a pathological feedback loop of functional dysregulation. This self-perpetuating axis drives progressive tissue destruction and systemic deterioration, fundamentally correlating with poor clinical outcomes (54).

Moreover, SLE is a systemic chronic inflammatory autoimmune disease characterized by multi-organ damage. BM-MSCs from SLE patients display prominent senescent characteristics, such as the increased SA-β-gal staining, disordered cytoskeletons, and slowed growth rates, which are mediated by multiple signaling pathways (55, 56). The osteogenic differentiation capacity of BM-MSCs from patients with SLE is reduced, which may participate in the pathology of osteoporosis in SLE patients (57). Moreover, the immunoregulatory functions of BM-MSCs in SLE are impaired, including the reduced proliferation rate and the increased apoptosis rate of BM-MSCs as well as the imbalance between Treg and Th17 (58). Altogether, MSCs in the SLE BMM are senescent, lose immunoregulatory capability, create a pro-inflammatory microenvironment, increase autoimmunity, and consequently further impair their own function, thus creating a vicious cycle that accelerates disease progression.

### 3.3 Dysregulated hematopoiesis

HSCs depend on the BMM to maintain quiescence and preserve self-renewal capacity. As both the cradle of immune cell production and the command center for immune system homeostasis, HSCs are particularly vulnerable to disruption under pathological conditions. Within the chronic inflammatory BMM of AIDs, HSCs undergo direct activation via Toll-like receptor (TLR) signaling or cytokine-mediated pathways, such as TNF-α. This chronic inflammatory stimulus causes a metabolic reprogramming of HSCs, altering their main energy metabolism from glycolysis to oxidative phosphorylation, which is followed by reactive oxygen species (ROS) accumulation (59–61). The resultant oxidative stress promotes excessive proliferation, accumulation of DNA damage, and ultimately leads to HSC apoptosis, senescence, or functional impairment (62). More importantly, the differentiation pathway of these inflammation-reprogramed HSCs is skewed toward a strong myeloid bias at the cost of lymphoid lineage development (63). Such lineage skewing plays a direct role in immune dyshomeostasis and has been found to contribute to the pathogenesis of numerous systemic autoimmune diseases, such as hematological autoimmune diseases and lupus erythematosus (64).

AIHA is primarily characterized by inadequate compensation of hematopoiesis and subsequent anemia, signifying ineffectiveness of the BM to respond adequately to heightened peripheral red blood cell destruction (65). Immune thrombocytopenia (ITP), a common hemorrhagic autoimmune disorder, is caused by autoimmune assaults on platelets and precursors of megakaryocytes. This dual mechanism leads to both accelerated platelet destruction and impaired platelet production, ultimately causing severe thrombocytopenia and clinical manifestations including petechiae, purpura, and bleeding complications (66). Megakaryocyte-biased progenitor cells of the BM of ITP patients are markedly reduced in number, and there is an imbalanced lineage differentiation with dysregulated progenitor cells. In particular, megakaryocyte-erythroid progenitors show a strong tendency to differentiate toward erythropoiesis at the cost of megakaryocyte development, leading to severely defective platelet production (67). At the same time, alterations in immune cell numbers, like increased B cells and reduced suppressive capacity, also contribute to the immune deviation (68). As thrombocytopenia persists and immune cell reserves become depleted, BM hematopoietic activity undergoes compensatory pathological activation; paradoxically, this compensatory response perpetuates and amplifies disease pathology rather than restoring homeostasis (67, 69).

Chronic inflammatory microenvironment and especially sustained type I interferon signaling in the BM of SLE patients abnormally stimulate HSCs and HSPCs in BM to exit quiescence and proceed to increased proliferation. The long-lasting activation leads to the build-up of DNA damage and the loss of their self-renewal ability, at the same time enhancing a strong bias toward myeloid and granulocyte lineages (64, 70). However, this myeloid skewing is accompanied by a differentiation block at the common myeloid progenitor stage, resulting in a net reduction of functional granulocyte progenitor populations despite the overall myeloid bias (50, 71). In addition to abnormalities in myeloid lineage, Gu et al. employed single-cell sequencing to reveal that B cell proportions in the BM of patients with SLE are already disturbed at early stages of development, particularly at the pre-B cell and immature B cell phases, indicating that BMM regulation of B lymphopoiesis is impaired from the earliest stages of lineage commitment (72–74). Taken together, these results suggest that HSC dysfunction drives abnormal developmental processes in both myeloid and lymphoid lineages from the earliest stages. This may be the underlying factor of the functional abnormalities in these immune cell groups throughout the lifespan in AIDs.

Beyond intrinsic HSC alterations, other cellular constituents of the BMM, such as BM-MSCs, endothelial cells, adipocytes, and nerve fibers, actively participate in regulating HSC quantity and function. As noted in the discussion above, BM-MSC-derived osteoblasts enhance the self-renewal, proliferation and differentiation of HSCs in several ways: through the production of extracellular matrix substrates including type I collagen and osteocalcin, and the expression and production of several key factors such as thrombopoietin (TPO), angiopoietin-1, osteopontin, stem cell factor (SCF), and CXCL12 (13, 75). As the core constituents of the vascular niche, sinusoidal endothelial cells serve dual functions. They not only form the structural channels through which HSCs transit between BM and circulation, but also directly support HSC survival and function through paracrine signaling (76, 77). Endothelial cells bidirectionally regulate HSC proliferation and dormancy via E-selectin and its antagonists, while continuously secreting SCF, CXCL12, and providing Notch signals to promote HSC colonization, quiescence, self-renewal, and survival (78, 79). Following BM injury, VEGFR2-dependent regeneration of sinusoidal endothelial cells represents a critical prerequisite for successful hematopoietic reconstitution (80, 81). Adipocytes, initially characterized as negative regulators of the BMM, exhibit an inverse correlation between their abundance and BM hematopoietic activity. Antagonizing adipocyte generation enhances hematopoietic recovery in clinical BM transplantation. However, recent studies have revealed more nuanced functions: adipocytes can produce hematopoietic-supportive factors including CXCL12, IL-8, and CSF3 to directly maintain HSC survival, and promote HSC regeneration and proliferation through SCF and adiponectin secretion (82–84). Regarding nerve fibers, the sympathetic nervous system within the BM directly governs circadian HSC mobilization by releasing adrenergic neurotransmitters from nerve endings, which regulate rhythmic CXCL12 expression. Sympathetic signals also modulate MSC function via β_3_-adrenergic receptors, thereby indirectly influencing HSC circadian migration patterns (85, 86). Additionally, unmyelinated Schwann cells ensheathing sympathetic nerves contribute to maintaining HSC quiescence in their vicinity by regulating activation of latent TGF-β-SMAD signaling (87).

Furthermore, immune cells, derived from HSCs, exert regulatory feedback effects on HSC function within the microenvironment. Tregs accumulate in specialized microenvironment compartments and provide direct protection to HSCs through co-localization, establishing an immune-privileged microenvironment that shields HSCs from immune-mediated attack (81, 88). B lymphocytes serve as an important source of acetylcholine, signaling to hematopoietic microenvironment cells to modulate immune cell production under both steady-state conditions and disease states (89). Macrophages anchor HSCs within the microenvironment by maintaining local CXCL12 chemokine levels and directly support HSC survival and post-transplantation engraftment (90). Neutrophils are dynamic messengers: their processes of senescence, migration, and subsequent clearance by macrophages transmit inter-organ and intercellular signals that indirectly modulate CXCL12 and G-CSF levels, thereby influencing HSC egress and overall microenvironment activity (91, 92). Taken together, these findings underscore that the BMM constitutes a complex and precisely orchestrated physiological system. Elucidating its working mechanisms and functional integration is of fundamental importance for deciphering disease pathogenesis and identifying novel therapeutic strategies.

### 3.4 Aberrant cytokines

The BMM is precisely regulated by a complex network of intrinsic and extrinsic factors. These regulatory elements profoundly influence immune system development and function by modulating microenvironment cellular composition and signaling pathways, thereby playing a critical role in determining disease susceptibility and shaping the clinical course of AIDs.

Patients with AIDs exhibit high levels of pro-inflammatory cytokines in the BMM including TNF-α and IFN-γ. In addition to damaging tissues directly, these inflammatory mediators cause epigenetic changes and metabolic changes in resident cells, altering energy metabolism, mitochondrial activity, and nutrient-sensing pathways. The resultant dysfunction of both immune and stromal cells perpetuates further inflammatory factor release, driving HSC metabolic reprogramming and myeloid-biased differentiation. It is a self-enhancing positive feedback loop that forms a vicious cycle, which sustains and aggravates the pathology of the disease (93). Acute inflammatory flare-ups and infections lead to the release of cytokines, particularly IFN, IL and TNF, which drive HSCs to exit quiescence and directly enter differentiation programs, rapidly replenishing the peripheral immune cell pool through proliferation and differentiation. In the process, HSCs are capable of adopting myeloid-biased differentiation programs to meet acute physiological needs (21, 94, 95). However, chronic or persistent inflammation leads to progressive HSC functional exhaustion, accelerated senescence, and sustained imbalance in immune cell composition, collectively increasing susceptibility to autoimmune diseases and potentially predisposing to hematological malignancies (96, 97).

Abnormal communication of chemokine and neuropeptide receptors, such as the CXCR4/CXCL12 axis, and sensory nerve-derived neuropeptides, such as CGRP, also interferes with BM vascular activity, adipocyte metabolism, and stromal remodeling. These intertwined dysregulations enhance pathological cytokine production, chronic tissue repair, and chronic inflammatory circuits in the microenvironment (98–100). Moreover, genetic and epigenetic processes are also inherent mechanisms which determine HSC function and the activity of the microenvironment in general. Transcription factors like RUNX1, GATA2 and TAL1 directly control gene programs that are necessary for HSC self-renewal and differentiation. Mutations affecting these genes or regulatory networks can precipitate congenital BM failure syndromes or predispose to leukemic transformation (101–103). Simultaneously, epigenetic modifications, such as DNA methylation and histone modifications, do not alter the DNA sequence but instead regulate gene activity. This regulation is vital in determining whether HSCs maintain stemness or commit to specific lineage trajectories, thereby fundamentally shaping HSC functional capacity and microenvironment homeostasis (104). Under homeostatic conditions, rather than aerobic respiration, HSCs principally use anaerobic glycolysis to maintain a low-metabolic quiescent state to minimize the damage caused by ROS, an adaptive mechanism. Disruption of this metabolic balance forces HSCs into the cell cycle, leading to progressive depletion of the stem cell pool (105). Maintenance of mitochondrial quality and prevention of dysfunction are critical for long-term HSC preservation, and functional integrity has been tightly coupled with the aging of HSCs. Moreover, excessive activation of nutrient-sensing pathways, such as mTOR, accelerates HSC exhaustion and compromises regenerative capacity (106, 107).

The higher incidence of AIDs in females, such as lupus, points to estrogen as a key predisposing factor (2). Emerging evidence suggests that this gender disparity may originate at the level of BM hematopoiesis. Estrogen has been demonstrated to enhance HSC self-renewal and expand HSC populations, exerting specific regulatory effects on HSPCs (108, 109). Studies utilizing selective estrogen receptor modulators such as tamoxifen have revealed that estrogen receptor alpha (ERα) activation induces apoptosis of short-term HSCs and multipotent progenitor cells, while simultaneously promoting quiescent long-term HSCs to enter the cell cycle. This twofold effect changes the expression profile of self-renewal genes and decreases HSC reconstitution capability in cases of stress (110). These findings suggest that the estrogen-enriched milieu in females may fundamentally “program” HSC behavior, rendering them more sensitive and responsive to immune challenges. Although this improved hematopoietic reactivity provides the benefits of an improved response to infection, there is a simultaneous rise in the threat of producing auto-reactive immunologic cell clones that provides a BM-focused explanation of the notable female disposition to AIDs. It is worth noting that there is no direct evidence available on the levels of estrogen in the BM supernatant of lupus patients and no research has been conducted so far to establish this possible relationship at the local microenvironmental level. It is expected that in future studies, this gap will be filled, as more mechanistic connections will be identified between sex hormone signaling, bone marrow hematopoiesis and AIDs pathogenesis.

### 3.5 Disrupted bone marrow–peripheral organ crosstalk

Beyond systemic AIDs such as SLE and RA, some autoimmune disorders display acute involvement of certain organ systems including the hematological, digestive, nervous, endocrine, and cutaneous systems. Although the clinical manifestations of these target organ-specific autoimmune diseases vary considerably, the underlying mechanisms of BMM pathology converge on some common themes: biased differentiation and developmental blockade within hematopoietic lineages, establishment of chronic inflammatory microenvironment, and metabolic or epigenetic reprogramming of microenvironment cellular functions.

The BMM plays a significant pathogenic role in AIDs by affecting the central nervous system. Multiple sclerosis (MS) is characterized by inflammatory demyelination of nerve fibers in the brain, spinal cord, and optic nerves. This process is accompanied by axonal injury and neurodegeneration, typically manifesting as acute attacks, relapsing course, and multifocal neurological involvement (111). The current research on MS, as well as animal model experimental autoimmune encephalomyelitis (EAE), has shown that there is a new pathology: the BM is an unexpected homing location of autoreactive T cells, which initially target the central nervous system and selectively migrate into the bone marrow space. Upon arriving there, they trigger HSPCs via the CCL5-CCR5 signaling axis, which results in biased differentiation into the myeloid lineage and orchestrates massive expansion of inflammatory cells, including neutrophils and monocytes (112). The newly produced myeloid cells in turn invade the central nervous system, significantly enhancing inflammatory reactions and demyelinating foci at early stages of the disease, thus creating a neuroinflammatory vicious cycle (113). Myasthenia gravis (MG) is a disease that is defined by a dysfunction of neuromuscular junction transmission dependent on both antibody-dependent and cellular immune responses which predominantly attack the neuromuscular junction acetylcholine receptors (114). Recent single-cell sequencing studies have uncovered molecular evidence of upregulated neutrophil maturation pathways in the BM of MG patients, indicating activation of the BMM accompanied by elevated levels of multiple pro-inflammatory cytokines (71). Refractory MG patients often achieve remission after HSCT, underscoring the pathogenic role of BM from a therapeutic perspective (115).

Abnormalities in the BMM also accompany AIDs affecting the endocrine glands. T1DM is a consequence of autoimmune destruction of pancreatic β cells, and the primary target organ is the pancreas. The chronic hyperglycemic environment characteristic of T1DM induces alterations in the BM cytokine milieu, leading to increased oxidative stress and endothelial dysfunction, which subsequently impair HSC self-renewal capacity and functional integrity (116, 117). Graves’ disease (GD) is caused by autoantibodies targeting thyroid-stimulating hormone receptor (TSHR), leading to hyperthyroidism and related clinical symptoms, such as hypermetabolism and excitation of the sympathetic nervous system (118). Aberrantly activated hematopoiesis in the GD BMM consists of stem cell expansion, the prominent infiltration of autoreactive T cells, the secretion of pro-inflammatory mediators (such as chemokine CCL5 and IL-6), and the degradation of anti-inflammatory cytokines (such as IL-10 and PGE_2_) (119).

The gut is a key organ of the immune system. In recent years, both the gut-brain axis and gut-metabolic axis have seen increased appreciation in the pathogenesis of diverse diseases including gastric cancer (120–122). In IBD, intestinal inflammation induces epigenetic and functional reprogramming of BM stem and progenitor cells through IL-23 and IL-17 signaling, which causes a state of emergency granulopoiesis characterized by massive neutrophil production to meet intestinal demands. This systemic hematopoietic activation establishes a self-reinforcing inflammatory state within the BMM. Such localized immune dysregulation serves as a primary driver of sustained chronicity and the characteristic relapsing-remitting clinical profile (123).

Cumulative clinical evidence and experimental evidence mentioned above point to the fact that the BMM is a widespread and constitutive pathology nexus in a variety of AIDs. Beyond merely providing inflammatory soldiers to their peripheral locations of autoimmunity, the BMM plays a significant role in disease progression, hematopoietic compensatory capacity, as well as susceptibility to recurrence. This new systems-level insight would offer a new theoretical paradigm and the BMM is an exciting new therapeutic target to intervene at the source level to treat autoimmune diseases.

## 4 Therapeutic strategies and future directions

Traditional management of AIDs has been based on immunosuppressants like glucocorticoids, azathioprine, and cyclosporine, which mainly regulate the disease process by suppressing the peripheral immune response. Although they are useful in the treatment of symptoms, they are associated with major side effects, such as infections, BM suppression, liver or kidney dysfunction (124–126). Prolonged medication use imposes substantial treatment burden on patients, and issues such as poor adherence further diminish therapeutic confidence, compromise quality of life, and contribute to unfavorable clinical outcomes (127, 128). Over the last several years, cellular therapy, especially HSC transplantation and MSC transplantation, has broadened the range of treatment and given fresh hope to the patients with refractory AIDs.

HSCT represents the most radical approach to resetting the central immune system. This strategy employs high-dose chemotherapy to ablate the aberrant immune system and partially remodel the diseased BMM, followed by reinfusion of autologous or allogeneic HSCs to reconstitute a new hematopoietic and immune system (129, 130). Accumulating evidence suggests that HSCT holds promise for achieving long-term remission and even potential “cure” for hematological AIDs, refractory MS and SLE (131).

MSC transplantation represents an important strategy for repairing and modulating the BMM. As a key functional component in the BMM, BM-MSCs possess multilineage differentiation potential and unique immunomodulatory properties. They can inhibit immune cell activation and induce regulatory T cells through cell-cell contact and paracrine pathways, so as to rebuild immune tolerance in the local microenvironment (132, 133). In addition, their ability to secrete growth factors, coupled with inherent homing capabilities, enables them to directly participate in the tissue repair and microenvironment regeneration. For example, the application of MSCs in AILD holds notable promise, yet it is also accompanied by formidable challenges. Experimental models have confirmed that BM-MSC transplantation effectively reduces liver injury, improves hepatic function, and enhances survival rates through multiple mechanisms, such as upregulating PD-L1 to inhibit IL-17, delivering exosomes carrying miR-223 to suppress NLRP3 inflammasome activation and restore Th17/Treg immune balance, and, following IL-35 gene modification, further inhibiting IFN-γ and FasL expression (134). These mechanistic insights provide a theoretical foundation for the clinical translation of BM-MSC therapy in AILD. Indeed, BM-MSC-based approaches have emerged as promising candidate strategies, with several clinical trials demonstrating safety and efficacy (134–136). After intravenous infusion, MSCs home to liver tissue and establish an anti-inflammatory microenvironment by inhibiting the production of inflammatory cytokines from liver-infiltrating T cells, macrophages and NK cells. Simultaneously, through TGF-β production, MSCs promote the expansion of immunosuppressive regulatory T cells (Tregs) and M2 macrophages, modulate CD4^+^ T cell activation, and inhibit the cytotoxicity of CD8^+^ T lymphocytes, NK and NKT cells, so as to alleviate liver immune injury (137). More recently, BM-MSC-derived exosomes have garnered attention as a promising cell-free therapeutic strategy due to low immunogenicity, ease of storage and potential for targeted delivery. Numerous preclinical studies have confirmed their immunomodulatory effect in liver diseases including AIH (138, 139). Nevertheless, the reliability, safety and efficacy of MSC-based therapies in liver disease treatment still lack robust evidence, and their clinical translation remains constrained by multiple factors (140–142). Accordingly, further research and exploration are required to advance MSC therapy development in AILD.

Moreover, allogeneic BM-MSC transplantation has proved to be a ray of hope in the treatment of refractory SLE, presenting itself with significant results in terms of improvement in disease activity index (SLEDAI), serological parameters, and urine creatinine clearance (143–145). However, in contrast, autologous BM-MSCs of lupus patients and model mice exhibit a senescent phenotype, impaired immunomodulatory capacity, and dysfunctional hematopoietic supportive function. Autologous BM-MSCs can also be reprogramed to partially regain their immunomodulatory properties (146, 147). In lupus models, rapamycin-pretreated umbilical cord-derived MSCs effectively inhibit B cell activation and autoantibody secretion through extracellular vesicle pathways, ultimately enhancing therapeutic efficacy (148). Infusion of BM-MSCs triggers neutrophil aggregation in recipient BM and generates an endogenous “extracellular vesicle (EV) storm” in the circulatory system via the TNF-α/ICAM-1/Rab11b axis. These EVs subsequently regulate Th17/Treg cell balance through the DHA/LILRB4/STAT5/STAT3 pathway, promoting immune tolerance and disease remission (145). At present, in various AIDs, BM-MSC transplantation has shown the potential to improve disease activity and preserve target organ function, with clinical application becoming increasingly widespread (149–152).

## 5 Conclusion

Under homeostatic conditions, the BMM precisely orchestrates HSC quiescence, self-renewal, and differentiation through an intricate network of cellular and non-cellular components. This finely tuned microenvironment actively participates in the establishment and maintenance of immune tolerance. However, in AIDs, persistent inflammatory stimuli disrupt homeostatic balance, inducing profound remodeling of the BMM. This pathological transformation manifests as HSC exhaustion, myeloid-biased differentiation, impaired immunomodulatory function of MSCs, metabolic and epigenetic reprogramming, and neural dysregulation. The resulting aberrant microenvironment not only fails to sustain normal immune homeostasis but actively fosters a microenvironment conducive to the generation of abnormal immune cells. This is closely linked to the sustained production of pro-inflammatory and autoreactive cell populations, thereby influencing the initiation and progression of diverse AIDs. Therefore, future therapeutic strategies necessitate multidimensional modulation of the microenvironment, shifting the paradigm from symptomatic management toward more fundamental interventions aimed at definitive restoration of immune tolerance.
